# Factors Influencing Dietary Patterns during Pregnancy in a Culturally Diverse Society

**DOI:** 10.3390/nu12113242

**Published:** 2020-10-23

**Authors:** Elisabet Fernández-Gómez, Trinidad Luque-Vara, Pablo José Moya-Fernández, María López-Olivares, Miguel Ángel Gallardo-Vigil, Carmen Enrique-Mirón

**Affiliations:** 1 Department of Nursing, Faculty of Health Sciences, Melilla Campus, University of Granada, C/Santander s/n, 52001 Melilla, Spain; elisabetfdez@ugr.es (E.F.-G.); triluva@ugr.es (T.L.-V.); 2 Department of Applied Economics, Faculty of Social and Legal Sciences, Melilla Campus, University of Granada, C/Santander s/n, 52001 Melilla, Spain; pjmoyafernandez@ugr.es; 3 Doctoral Degree School, Melilla Campus, University of Granada, C/Santander s/n, 52001 Melilla, Spain; 4 HUM-358 Research Group, Department of Research and Diagnostic Methods in Education, Faculty of Education and Humanities, Melilla Campus, University of Granada, C/Santander s/n, 52001 Melilla, Spain; magvigil@ugr.es; 5 HUM-613 Research Group, Department of Inorganic Chemistry, Faculty of Health Sciences, Melilla Campus, University of Granada, C/Santander s/n, 52001 Melilla, Spain; cenrique@ugr.es

**Keywords:** dietary patterns, pregnancy, sociodemographic factors, pregnancy-related factors, lifestyle, factor loadings

## Abstract

The aim of this study was to identify dietary patterns in pregnant women and to assess the relationships between sociodemographic, lifestyle-related, and pregnancy-related factors. This is a descriptive, correlational study involving 306 pregnant women in Melilla (Spain) in any trimester of pregnancy. A validated food frequency questionnaire was used. Dietary patterns were determined via exploratory factor analysis and ordinal logistic regression using the proportional odds model. Three dietary patterns were identified: Western, mixed, and prudent. Sociodemographic, lifestyle-related, and pregnancy-related factors influencing dietary quality were established. The Western dietary pattern was considered the least recommended despite being the most common among women who live in Melilla (*p* = 0.03), are Christian (*p* = 0.01), are primiparous women (*p* < 0.001), and are in their first or second trimester (*p* = 0.02). Unemployed pregnant women were also more likely to have a less healthy dietary pattern (*β* = −0.716; *p* = 0.040). The prudent dietary pattern, the healthiest of the three, was most commonly observed among Muslim women (*p* = 0.01), women with more than two children (*p* < 0.001), and women in the third trimester of pregnancy (*p* = 0.02). Pregnant women who engaged in no physical activity or a low level of physical activity displayed a mixed pattern (*p* < 0.001). This study provides evidence on the factors influencing dietary patterns during pregnancy and suggests that more specific nutrition programmes should be developed to improve the nutritional status of pregnant women.

## 1. Introduction

A range of dietary factors contribute to the development of conditions such as heart disease, stroke, type 2 diabetes, and cancer [1,2,3].

From the end of the 19th century, the Spanish diet gradually evolved to meet the population’s energy and nutrient requirements, although meeting these needs remained more difficult for minors, adult women, and pregnant women. However, at the end of the 20th century, as in other countries, energy intake increased, becoming excessive, unbalanced, and deficient in terms of the main micronutrients [4].

Pregnancy is a critical, vulnerable state, where maternal nutrition and lifestyle constitute the main influences on the health of both mother and newborn. During pregnancy, the need for nutrients, especially micronutrients, increases compared to other stages of life, leading in turn to higher nutritional requirements [5,6]. Inadequate maternal nutritional intake during pregnancy may lead to negative short- and long-term health consequences for both the mother and her child [7,8,9]. In the scientific literature, it is widely recognised that environmental factors such as diet during pregnancy can contribute to the development of certain diseases among offspring [10,11,12,13,14] as well as the occurrence of metabolic alterations (overweightness or obesity) in pregnant women, especially in early pregnancy, which increase the probability of obesity in their future children [15,16].

What foods should be eaten, in what amounts, and how often are questions for which answers will enable the provision of a concise nutritional assessment and appropriate guidance during pregnancy [17].

Recent studies have shown that diets with a higher intake of fruits, vegetables, pulses, and fish lead to positive outcomes during pregnancy [18,19]. Generally speaking, greater adherence to the Mediterranean diet during pregnancy can protect against excessive cardiometabolic risk for offspring [20].

Most studies on this topic have focused on analysing individual nutrients, while epidemiological research highlights the importance of assessing the effect of dietary quality on overall health by establishing dietary patterns. This approach has several advantages. For example, it takes into account the interactions between food components [21] and identifies statistically significant associations by random chance. The effect of a single food component may also not be large enough to be detected. This is why dietary patterns allow us to analyse sociocultural or environmental aspects and to identify the cumulative effects of the nutrients in a single pattern.

Foodstuffs are not consumed in isolation, and individuals eat foods that include a combination of nutrients. Dietary patterns give a broader overview of food and nutrient consumption, so they may be more predictive of the risks of developing certain diseases than individual foods or nutrients [22].

In general, studying dietary patterns constitutes a more comprehensive approach and has proven very useful in providing significant results to a given population [23,24]. Studying dietary patterns during pregnancy is one of the most suitable approaches for demonstrating the effect of diet on maternal and neonatal health [25].

Current patterns in preconception care emphasise that certain lifestyle factors, particularly nutrition, play an important role in pregnancy [17]. Pregnant women’s nutritional knowledge, among other factors such as social or cultural factors, can influence their food intake [26]. Religion is one cultural factor that conditions the diet of believers [27,28], as most religions set rules concerning the intake of certain foods, distinguish between pure and impure foods, determine times for fasting, etc. [29]. Similarly, psychological disorders such as depression, anxiety, or stress may also influence dietary choices during pregnancy [30,31,32].

For all these reasons, it is of paramount importance to determine the factors that may influence the adoption of certain types of dietary patterns. Several authors have recommended that further studies be conducted to specifically assess different factors as potential determinants [24], as the consequences of inadequate nutrition put not only women but also their infants at risk of poorer health outcomes for the rest of their lives. The aim of this research is to promote health and to develop effective interventions for this vulnerable population.

This study seeks to determine dietary patterns in pregnant women and to assess the relationships between sociodemographic factors (place of residence, age, religion, level of education, marital status, socioeconomic status (level of income per month), and employment status), lifestyle-related factors/health-related behaviours (alcohol consumption, level of physical activity, prepregnancy body mass index (BMI), and supplement consumption), and pregnancy-related factors (parity, trimester, and attendance at antenatal classes).

## 2. Materials and Methods

### 2.1. Study Design and Sample

This was a diagnostic study of a descriptive-correlational nature, using a cross-sectional design. Data were collected through questionnaires, which were administered in person to a sample of pregnant women at the Women’s Care Unit in the autonomous city of Melilla, Spain, when attending the unit for antenatal care. Intentional (or convenience) sampling was used.

The inclusion criteria were as follows: being pregnant in any of the trimesters, not having undergone assisted reproductive technologies (ARTs), not having a high-risk pregnancy, and not following any special diet. The mean number of annual births in the city where the study was being conducted (926.16 ± 104.93) was also taken into account. The sample was made up of a total of 306 pregnant women.

The study sample was recruited from March 2018 to February 2019. None of the pregnant women refused to participate in the study. According to the Spanish Healthcare System, the mean number of annual births reported in Melilla during the previous 18 years was 926.16 ± 104.93. This figure was used to estimate a representative sample size (272), which was increased to 306 to ensure the representativeness of the sample.

### 2.2. Assessment of Dietary Patterns

#### 2.2.1. Food Frequency Questionnaire

The participants were assisted in person by a trained professional to complete an adapted version of the Cuestionario de Frecuencia de Consumo de Alimentos or Food Frequency Questionnaire (FFQ) by Trinidad Rodríguez et al. (2008) [33]. The questionnaire asks about the number of times per day, per week, and per month they had consumed foods and drinks from a list of 39 foods and 6 drinks, with a total of 45 foods and drinks, in the last month.

A second questionnaire was also used to collect sociodemographic data; data regarding lifestyle, such as level of physical activity, prepregnancy BMI, supplement consumption, and intake of alcoholic drinks; and data related to pregnancy itself, such as parity, trimester, and attendance at antenatal classes. The time required to complete the full questionnaire ranged from 15 to 20 min.

#### 2.2.2. Preprocessing of Dietary Data

To quantify the frequency categories and to standardise the intakes for one day, the data were translated into the following numerical values: (a) 0 (never), (b) 0.07 (1–3 times per month), (c) 0.15 (once a week), (d) 0.45 (2–4 times a week), (e) 0.8 (5–6 times a week), (f) 1 (once a day), (g) 2.5 (between 2 and 3 times a day), (h) 5 (5 times a day), and (i) 6 (more than 6 times a day).

In the post hoc identification of dietary patterns, the 45 items in the FFQ were regrouped into 21 food groups based on similarity of nutrient profiles and comparable use (Table 1). To standardise the results, the mean intake values for each group were considered.

### 2.3. Sociodemographic, Lifestyle-Related, and Pregnancy-Related Factors

The following sociodemographic variables were considered: age, place of residence, marital status, religion, level of education, employment status, and level of income per month. The following lifestyle-related variables were taken into account: alcohol consumption, level of physical activity, prepregnancy BMI, and supplement consumption. Finally, the pregnancy-related factors analysed were parity, gestational trimester, and attendance at antenatal classes. The response options for level of physical activity were none/low, moderate, and high. To define physical activity, we used the International Physical Activity Questionnaire (IPAQ), whereby a moderate level of physical activity may mean the following: 3 or more days of vigorous physical activity at least 20 min per day, 5 or more days of moderate physical activity and/or walking at least 30 min per day, or 5 or more days of any combination of walking and moderate or vigorous physical activity. Lower levels of physical activity are considered to be low or no physical activity, whereas higher levels are considered to be high physical activity. In order to define alcohol consumption and attendance at antenatal classes, the response options were yes, no, and sometimes.

### 2.4. Statistical Analysis

All statistical analyses were performed using the SPSS Statistics for Windows, Version 24.0 (International Business Machines Corporation, IBM, Armonk, NY, USA). The statistical significance threshold for the results was set at *p* < 0.05. A descriptive analysis of sociodemographic variables, lifestyle-related factors, and pregnancy-related factors was carried out.

An exploratory factor analysis was conducted to identify dietary patterns based on food consumption data [21,22,24,35]. This approach was informed by the observation that scores for the consumption of some foods are correlated, and thus, statistical factor analyses can be used to express the total variance of dietary questionnaire scores in terms of some latent variables (factors). The factors were identified using the minimum sum of squared residuals, followed by varimax rotation for interpretation.

Of the total 21 food groups, 2 groups (alcoholic drinks and diet drinks) had to be discarded as their presence prevented factorial analysis. The factor loadings for the remaining 19 food groups were calculated. Factor loadings represent the extent to which the food group is related to a particular factor. A positive factor loading for a food group means that the factor represents preference for a food group, while a negative factor loading means that the factor represents avoidance of that food group (less than the mean frequency of consumption). Factor loadings greater than 0.2 were considered to represent an interpretable association regarding the corresponding factor and were thus used to describe and label the dietary patterns. Factor loadings below 0.2 were discarded [36].

Of the total number of factors, two were assumed based on the variance explained by the successive components of the food score matrix. These two factors explained 22.65% of the variance (Table 2). This percentage is comparable to and even slightly higher than those reported in other studies in this field [24,36,37].

The labels given to the dietary patterns (prudent and Western) do not perfectly describe each underlying pattern, but they are helpful when analysing and discussing the results. The prudent dietary pattern was characterised by a high consumption of fruits, nuts, vegetables, pulses, cereals, fish, and poultry; by a moderate consumption of dairy products and cheese; and by a low consumption of red meat and cold cuts in particular. By contrast, the Western dietary pattern was characterised by high consumption of red meat and cold cuts, potatoes, cakes and pastries, chocolate, and sugary drinks and by a very low intake of pulses, fruits, and cereals (Table 2). These dietary patterns were based on the Mediterranean diet and the Spanish recommendations for pregnant women.

A new variable was used as an overall measure of the participants’ relative tendency towards prudent or Western dietary patterns. This variable was calculated as the difference between the total recommended daily intake for each food group and the total daily intake score for all food groups. The results obtained were classified into three categories based on two cutoff points: percentiles 33.3 and 66.6. Negative values were classified as “Western”, intermediate or moderate values in the middle of the frequency distribution were classified as “mixed”, and the most positive values were classified as “prudent”.

The association between sociodemographic, lifestyle-related, and pregnancy-related determinants and dietary patterns in each of the three dietary categories was assessed using Pearson’s chi-squared test.

Finally, an ordinal logistic regression analysis was conducted to identify the relationships of sociodemographic, lifestyle-related, and pregnancy-related variables with dietary patterns during pregnancy using the proportional odds model, which is frequently used with health questionnaires.

### 2.5. Ethical and Legal Considerations

The confidentiality of the data and the anonymity and privacy of the participants were preserved at all times, in compliance with the Spanish Organic Law 15/1999, of the 13th of December, on personal data protection.

The ethical principles set out in the Declaration of Helsinki were also observed. All participants were therefore informed of the purpose of this study and participated voluntarily having signed an informed consent form.

Approval was obtained from the Directorate of Health Care Management of the Health Area of Melilla with reference: PSVG/ppg on 18 October 2017, on which the Women’s Care Unit depends, as well as the midwives of the Unit.

## 3. Results

### 3.1. Characteristics of the Study Population

All the sociodemographic, lifestyle-related, and pregnancy-related characteristics of the sample are listed in Table 3.

The mean age of the sample was 29.92 ± 5.51 years old, ranging from 18 to 43 years old. Most (83.3%) of the participants were living in Melilla, while the remaining participants (16.7%) came from the other side of the border; 86.6% were married or in a relationship. Just over two thirds (67.6%) reported being Muslims, 28.4% identified as Christians, and the remaining participants had other religions or beliefs. Employment rates were as follows: 47.1% were employed, 20.3% were unemployed, and only 1.7% were studying or on sick leave. For 38.2%, it was their first pregnancy, 12.1% had more than two children, and the rest had between 1 and 2 children; 32.7% were in their first trimester of pregnancy, 34.3% were in their second, and 33% were in their third. Regarding prepregnancy BMI, just under half of the participants (43.8%) had a normal weight, while the others deviated from the norm: 30.7% were overweight, 21.2% were obese, and only 4.2% were underweight. Finally, most of the women (82.7%) engaged in no physical activity or a low level of physical activity, whereas 17.3% had a moderate level of physical activity. The most commonly used supplements were those containing folic acid, iron, iodine, and vitamin B12. Almost all (99.7%) of the sample did not consume alcoholic drinks.

### 3.2. Diet during Pregnancy

The results of the FFQ are shown in Table 4.

With regard to foods consumed daily, bread stands out as one of the most frequently consumed foods, with 63.4% of participants eating it from 2 to 3 times a day, followed by coffee/tea (39.2%), with the same frequency of consumption. Of the foods consumed only once a day, it is worth mentioning dairy products (32.7%).

With respect to foods consumed weekly, just over half of pregnant women consume 2–4 eggs per week (56.5%), although a not inconsiderable percentage (13.7%) do not consume eggs on a weekly basis or never eat them. The most frequent consumption of pulses and white meat is 2–4 times a week; 48.3% eat red meat, 40.8% eat cheese, and 62.1% eat fish once a week, while 35.9% never eat nuts.

Regarding foods consumed occasionally by pregnant women, 55.6% do not consume cold cuts compared with 27.1% who consume them from 2 to 4 times a week; 33% do not consume cakes or pastries, while 36.3% do so on a weekly basis, as is the case with sugary drinks (32.4%); and 30.4% report eating chocolate between 2 and 4 times a week, while 35.3% never eat it.

### 3.3. Sociodemographic, Lifestyle-Related, and Pregnancy-Related Determinants of Dietary Patterns

The Western dietary pattern was most commonly found among women living in Melilla (*p* = 0.03), who are Christians (*p* = 0.01), are primiparous women (*p* < 0.001), and are in their first or second trimester of pregnancy (*p* = 0.02) (Table 5). On the other hand, the prudent dietary pattern was most often seen in Muslim women (*p* = 0.01), women with more than 2 children (*p* < 0.001), and women in their third trimester of pregnancy (*p* = 0.02). Pregnant women who engaged in no physical activity or a low level of physical activity displayed a mixed pattern (*p* < 0.001).

With regard to BMI, the group of pregnant women classified as obese exhibited a prudent dietary pattern to a greater extent (41.4%) than those who were overweight, who preferred a mixed dietary pattern (38.3%). Pregnant women with normal weight were equally distributed across the three dietary patterns.

Regarding age, 60% of pregnant women under 19 years old had a mixed pattern, whereas 50% of those over 40 followed the prudent dietary pattern.

Table 6 shows the sociodemographic, lifestyle-related, and pregnancy-related determinants of dietary pattern during pregnancy according to the ordinal linear regression model. The categories of the dependent variable, the dietary pattern, were ordered from lowest to highest based on their degree of healthiness. Of the variables studied, four showed a significant association with the dietary pattern followed during pregnancy: religion, employment status, number of children, and trimester of pregnancy. Specifically, Muslim women were more likely to follow a healthier dietary pattern (*β* = 0.680; *p* = 0.038), as were pregnant women who had more than two children (*β* = 1.002; *p* = 0.022) and those who were in their third trimester (*β* = 0.819; *p* =0.005). Unemployed pregnant women were more likely to adopt an unhealthier, less recommended dietary pattern (*β* = −0.716; *p* = 0.040).

## 4. Discussion

The present study identified three different dietary patterns (prudent, mixed, and Western) based on dietary data collected during all three trimesters of pregnancy. Through ordinal regression analysis, clear sociodemographic, lifestyle-related, and pregnancy-related determinants of dietary quality were established. Being Muslim, having more than two children, and being in the third trimester of pregnancy were significant factors of a healthier, more advisable diet during pregnancy. However, being unemployed during pregnancy was significantly associated with a less healthy dietary pattern.

Dietary pattern approaches are most often used to assess the quality of the diet as a whole. Firstly, the degree of compliance with dietary guidelines needs to be determined to define a diet before the predefined food patterns are assessed as either promoting or being detrimental to health. Several recent studies associate certain factors with what are known as dietary patterns [38,39,40]. In this study too, a number of factors were associated with dietary patterns.

As in this study, several other studies associate dietary quality with influencing factors during pregnancy [35,41,42,43,44,45]. Dietary pattern analyses take into account a diet as a whole and allow data from observational studies to be interpreted into eating behaviours that can be used to inform public health guidelines and recommendations [46].

For years, two procedures have been used to study dietary patterns based on prior knowledge, i.e., dietary requirements [47] and statistical tests such as principal components analysis [48]. Exploratory factor analysis, which was used in the present study, and principal components analysis are two similar techniques used to create dietary patterns. Numerous studies also conduct exploratory factor analysis to identify major dietary patterns [21,22,35,49,50,51,52].

Several studies use multiple linear regression models to analyse associations between lifestyles, sociodemographic variables, and different dietary patterns [24,53]. However, when the dependent variable under study is of an ordinal nature, it is not appropriate to use multiple linear regression models and other techniques must therefore be used [54]. A commonly used technique is the logit version of the ordinal regression model, also known as the proportional odds model [55]. This was the method used in this study, and its main advantage lies in the simplicity of interpreting its coefficients compared to other alternatives. Furthermore, this type of technique is preferable to the dichotomisation of the dependent variable, which results in a loss of information and efficiency when analysing the results [56]. The proportional odds model is frequently used with health-related questionnaires, and several recent studies have associated dietary patterns with various factors using this model [57,58,59].

Common tools for collecting dietary data are 24-h quantitative intake recalls, dietary records, and FFQs [60]. FFQs are widely used to generate dietary patterns [25,35,61,62]. In the case of the present study, the method used to collect dietary data was the FFQ by Trinidad Rodríguez et al. (2008) [33]. It should be noted that the different FFQs used to collect daily, weekly, or monthly data on the frequency of consumption of a set of foods are very similar to one another [63,64].

Most analyses determining dietary patterns indicate that whole grains, fruits, vegetables, pulses, and fish correspond to health-promoting dietary patterns, while refined grains, processed meats, and cakes and pastries are characteristic of a less healthy diet [65].

Other dietary patterns display regional and cultural influences on dietary intake. Examples include the common Brazilian pattern, with consumption of rice or pasta and beans with beef, chicken, eggs, margarine, coffee, and packaged fruit juices, as reported in a study by Hoffmann et al. (2013) [66], or the pattern found in the southern United States, where eggs, cooked cereals, peaches, corn, fried fish, vegetables, cabbage, sweet potatoes, liver, oxtail, pork, and fresh fruit juices are consumed, as reported by Völgyi et al. (2013) [67].

Dietary patterns are specific to different populations, although they may vary with age, socioeconomic status, ethnicity, culture, and the availability of different foods. There are marked differences in the dietary profiles of different countries in Eastern and Western Europe [24,68,69].

In a systematic review, Doyle et al. (2017) established that the most frequently studied sociodemographic factors are level of education, age, ethnicity, nationality, level of income per month, cohabitation, and occupation as well as place of residence as an environmental factor. The most commonly used lifestyle-related factors were prepregnancy BMI, tobacco use, and level of physical activity. Other factors, such as the consumption of supplements, alcohol, and caffeine, were assessed less frequently. Parity was the most frequently assessed pregnancy-related determinant [35]. This study took all of these factors into account, in addition to the gestational trimester and attendance at antenatal classes. The only factor not included was tobacco use.

The different dietary patterns identified in pregnant women have been found to be associated with sociodemographic determinants. Indeed, higher socioeconomic status is associated with a healthier diet in this group of women [66,70,71]. This study corroborates these results inversely, as unemployed pregnant women exhibit an unhealthier, less recommended dietary profile.

The diets of women living in urban areas are more likely to be of poorer quality [72,73]. The present study echoes these findings, with the less healthy Western dietary pattern more commonly found among women living in the city.

Dietary patterns in particular differ across populations and cultural contexts and are sometimes difficult to interpret [17]. As previously mentioned, religion falls under culture and conditions what individuals eat. The present study reflects a clear influence between religion and the dietary pattern exhibited. Significant associations were observed in Muslim pregnant women, who showed a preference for the prudent dietary pattern.

Age, level of education, and lifestyle influence dietary quality [61]. Some studies report that older, more educated women who are regularly physically active are more likely to follow healthier dietary patterns [35,43,74,75]. This is because, in addition to sociodemographic factors, lifestyle also influences dietary patterns. Previous studies have reported the association of dietary patterns with prepregnancy BMI [25,41,73,76,77]; however, other studies do not report this association [66]. There is evidence that pregnant women with lower BMIs are associated with a healthier diet [78]. In the present study, pregnant women with obese BMIs are more likely to follow a prudent or healthy dietary pattern, as another recent study shows [24]. This may be because pregnancy has been described as a period of increased motivation for behavioural change [79].

No significant associations with dietary patterns were found regarding the remaining variables, i.e., age, level of education, and the rest of the lifestyle-related variables.

Women who used supplements during pregnancy did not differ in their dietary choices from those who did not use them. Fowler et al. (2012) [80] obtained similar results. However, Laraia et al. (2007) [81] found that pregnant women who had used supplements before pregnancy followed a healthier diet. It should be noted that 92.2% of the women in this study took supplements, as they are highly recommended by specialists, which may have resulted in this lack of association. This was also the case in a study by Wesolowska et al. (2019) [24].

No statistically significant impact of alcohol consumption on dietary pattern during pregnancy was found in this study, although some studies have shown that alcohol consumption is a determinant of unhealthy dietary patterns [35,49,51], while others found no significant correlation [24].

Regarding pregnancy-related factors, we found that parity and gestational age were associated with dietary patterns. Women with more than two children and those in the third and final trimester of pregnancy display a healthier dietary profile. In previous research, parity was also associated with compliance with guidelines [80] and dietary quality [43]. However, in other studies, the association between parity and diet quality was either inverse [74,82] or did not appear to have any influence on healthy eating habits [73]. In a cohort study in Spain, the pattern considered the healthiest was positively associated with gestational age at 10 and 38 weeks [53]. A similar phenomenon can be seen in the present study, where pregnant women with a gestational age around the third trimester are those with a more advisable dietary pattern.

In view of the results obtained, we recommend that proposals for intervention in the nutritional education of pregnant women and women in the preconception stage should address not only nutritional aspects but also participants’ sociodemographic characteristics, so that more personalised training can be delivered with more effective outcomes.

The Spanish Ministry of Health, Social Services, and Equality (2014) notes that experts recommend health education for pregnant women to encourage them to adopt healthy eating habits [83]. There is a lack of knowledge and considerable concern regarding different eating parameters among pregnant women. This is why pregnancy is an ideal stage for providing nutritional education [84].

Numerous studies have sought to improve nutritional knowledge, attitudes, and habits during pregnancy through nutritional interventions, concluding that nutritional education had positive, effective repercussions [85,86,87,88,89,90,91,92,93].

The International Federation of Gynecology and Obstetrics stresses the importance of a varied, healthy diet and encourages the adoption of healthy eating habits before and during pregnancy [94]. Other institutions, such as the Spanish Nutrition Foundation (2013) and the Spanish Ministry of Health, Consumer Affairs, and Social Welfare (2019), also set out nutritional recommendations based primarily on the following: improving nutritional quality; regulating calorie intake; adapting to recommended weight gain; increasing consumption of fruit, vegetables, and leafy vegetables; taking adequate supplementation; exercising daily; and avoiding foods high in fat and sugars, alcoholic drinks, and stimulants such as coffee or tea [95,96].

One of the strengths of this study is that all data from the sample were collected in person by a qualified nurse to ensure that they were as accurate as possible. The study’s inclusion criteria (healthy women without previously existing conditions such as diabetes or high blood pressure) is also relevant, as this may have resulted in the pregnant women selected not requiring a special diet. Furthermore, drawing on a systematic review that examines studies focusing on the determinants of dietary patterns during pregnancy [35], this research not only takes into account sociodemographic variables but also looks at lifestyle- and pregnancy-related factors, which could help to more accurately assess dietary predictors.

Regarding the limitations of this study, it should be noted that several authors have analysed the effect of reducing the number of items by grouping foods together, concluding that it does not affect the identification of dietary patterns. However, this would have an effect on the percentage of variance explained [97]. Most of the women (82.7%) performed no physical activity or a low level of physical activity, whereas 17.3% of them performed a moderate level of physical activity, which may help to explain the lack of association of physical exercise with dietary patterns. It should also be mentioned that the only factor not included in this study was tobacco use, which is usually taken into account in other researches. In addition, as discussed above, dietary patterns differ across populations and cultural contexts, making it rather difficult to draw comparisons.

## 5. Conclusions

Maintaining a healthy, recommended dietary pattern during pregnancy plays a key role in optimising maternal and child health. Sociodemographic determinants and lifestyle-related and pregnancy-related factors influence dietary patterns during pregnancy, which suggests that the modification of eating behaviours and the development of more specific nutrition programmes to improve the nutritional status of pregnant women should be promoted.

Since dietary patterns vary across countries, it is important to identify country-specific dietary patterns that may be associated with the aforementioned results. Therefore, analysing dietary patterns in different populations is key to developing relevant prevention and health promotion strategies. Finally, these results should be used to inform educational programmes and interventions focusing on healthy diet recommendations during pregnancy.

## Figures and Tables

**Table 1:** Description of the food groups.

Food Groups	Number of Items	Foods
Dairy products	3	Milk, yoghurt, and homemade ice cream
Cheese	2	Fresh cheese, and cured or semi-cured cheese
Eggs	1	Eggs
White meat	1	Chicken and turkey
Cold cuts	1	Cold cuts and paté
Red meat	2	Red meat (beef, pork, and lamb) and minced meat
Fish	3	White fish, blue fish, and seafood
Vegetables	3	Salads, greens, and vegetable garnish
Fruit	3	Citrus fruits, fruit, and fresh fruit juice
Nuts	1	Nuts (almonds, peanuts, hazelnuts, walnuts, etc.)
Pulses	1	Pulses (lentils, chickpeas, pinto or haricot beans, and cooked peas)
Cereals and pasta	2	Rice and pasta (spaghetti, noodles, macaroni, etc.)
Potatoes	2	Potatoes and crisps
Bread	1	Bread
Cakes and pastries	7	Marie biscuits, chocolate biscuits, doughnuts, muffins, cakes, pastries, and canned fruit
Chocolate	1	Chocolate
Sugary drinks	2	Sugary drinks and packaged fruit juices
Coffee and tea	2	Coffee and tea
Wine and beer	2	Wine (red wine, white wine, and rosé) and beer
Alcoholic drinks	1	Brandy, gin, rum, whisky, vodka, and 40-proof spirits
Diet drinks	1	Sugar-free drinks

Adapted from Ciprián et al. (2013) [34].

**Table 2:** Factor loadings for the different food groups for each dietary pattern (*N* = 306).

Food Groups	Prudent Pattern	Western Pattern
Dairy products	0.081	−0.245
Cheese	0.080	−0.030
Eggs	0.205	−0.004
White meat	0.138	−0.035
Cold cuts	−0.372	0.044
Red meat	0.023	0.300
Fish	0.573	0.253
Vegetables	0.616	−0.207
Fruit	0.555	0.021
Nuts	0.581	−0.012
Pulses	0.278	0.080
Cereals and pasta	0.639	0.057
Potatoes	0.049	0.783
Bread	0.079	0.153
Cakes and pastries	−0.051	0.732
Chocolate	−0.067	0.315
Sugary drinks	−0.083	0.673
Coffee and tea	0.193	0.067
Wine and beer	−0.089	−0.103
Variance explained (%)	10.093	12.557

**Table 3:** Sociodemographic, lifestyle-related, and pregnancy-related variables (frequencies and percentages, *N* = 306).

	Variables	Frequency (%)
Living in Melilla	Yes	255 (83.3)
	No	51 (16.7)
Age	<19 y.o.	5 (1.6)
	20–39 y.o.	289 (94.4)
	>40 y.o.	12 (3.9)
Marital status	Single	38 (12.4)
	Married/In a relationship	265 (86.6)
	Separated/divorced	3 (1)
Religion	Muslim	207 (67.6)
	Christian	87 (28.4)
	Other	12 (3.9)
Level of income per month	<€500	12 (3.9)
	€501–1000	101 (33)
	€1001–2000	113 (36.9)
	€2001–5000	74 (24.2)
	>€5001	6 (2)
Level of education	No education	23 (7.5)
	Primary and secondary education	112 (36.6)
	A levels/Higher vocational training	93 (30.4)
	University/Postgraduate education	78 (25.5)
Employment status	Household chores	95 (31)
	Employed	144 (47.1)
	Unemployed	62 (20.3)
	Student	3 (1)
	On sick leave	2 (0.7)
Parity	None	117 (38.2)
	1–2	152 (49.7)
	>2	37 (12.1)
BMI	Underweight (<18.5)	13 (4.2)
	Normal weight (18.5–24.9)	134 (43.8)
	Overweight (25.0–29.9)	94 (30.7)
	Obese (≥30)	65 (21.2)
Level of physical activity	None/Low	253 (82.7)
	Moderate	53 (17.3)
	High	0 (0)
Trimester	First trimester	100 (32.7)
	Second trimester	105 (34.3)
	Third trimester	101 (33)
Attendance at antenatal classes	Yes	119 (38.9)
	No	176 (57.5)
	Sometimes	11 (3.6)
Supplement consumption	Yes	282 (92.2)
	No	24 (7.8)
Alcohol consumption	Yes	2 (0.7)
	No	304 (99.3)

y.o.: years old; BMI: Body mass index.

**Table 4:** Consumption of food groups (frequencies and percentages).

Food Groups	Never	1–3 Times a Month	Once a Week	2–4 Times a Week	5–6 Times a Week	Once a Day	2–3 Times a Day
Dairy products	11 (3.6)	6 (2)	73 (23.9)	40 (13)	76 (24.8)	100 (32.7)	
Cheese	50 (16.3)	26 (8.5)	125 (40.8)	85 (27.8)	11 (3.6)	9 (2.9)	
Eggs	27 (8.8)	15 (4.9)	62 (20.3)	173 (56.5)	24 (7.8)	5 (1.6)	
White meat	13 (4.2)		53 (17.3)	205 (67)	29 (9.5)	6 (2)	
Cold meats	170 (55.6)		32 (10.5)	83 (27.1)	13 (4.2)	8 (2.6)	
Red meat	48 (15.7)	31 (10.1)	148 (48.3)	79 (25.8)			
Fish	51 (16.7)	40 (13)	190 (62.1)	25 (8.2)			
Vegetables	13 (4.2)	9 (2.9)	133 (43.5)	110 (35.9)	13 (4.2)	28 (9.2)	
Fruit	6 (2)	4 (1.3)	64 (20.9)	128 (41.8)	75 (24.5)	29 (9.5)	
Nuts	110 (35.9)		48 (15.7)	111 (36.3)	20 (6.5)	17 (5.6)	
Pulses	24 (7.8)		103 (33.7)	173 (56.5)	5 (1.6)	1 (0.3)	
Cereals and pasta	11 (3.6)	29 (9.5)	200 (65.3)	66 (21.6)			
Potatoes	15 (4.9)	26 (8.5)	159 (52)	101 (33)	5 (1.6)		
Bread	18 (5.9)		8 (2.6)	32 (10.5)	39 (12.7)	15 (4.9)	194 (63.4)
Cakes and pastries	101 (33)	88 (28.7)	111 (36.3)	6 (2)			
Chocolate	108 (35.3)	8 (2.6)	57 (18.6)	93 (30.4)	19 (6.2)	12 (3.9)	9 (2.9)
Sugary drinks	101 (33)	33 (10.8)	99 (32.4)	55 (18)	9 (2.9)	9 (2.9)	
Coffee and tea	151 (49.3)					24 (7.8)	120 (39.2)
Wine and beer	293 (95.7)	10 (3.3)	3 (1)				

Note: As per the groups detailed in Table 1. Alcoholic and diet drinks have been excluded due to low consumption levels among participants.

**Table 5:** Sociodemographic, lifestyle-related, and pregnancy-related variables and dietary patterns (*N* = 306) *.

Variables	Western Pattern	Mixed Pattern	Prudent Pattern	*p*
Living in Melilla				
Yes (*n* = 255)	93 (36.5)	80 (31.4)	82 (32.2)	0.031
No (*n* = 51)	9 (17.6)	22 (43.1)	20 (39.2)	
Age				
<19 y.o. (*n* = 5)	2 (40.0)	3 (60)	0	0.366
20–39 y.o. (*n* = 289)	97 (33.6)	96 (33.2)	96 (33.2)	
>40 y.o. (*n* = 12)	3 (25)	3 (25)	6 (50)	
Religion				
Christian (*n* = 87)	40 (46)	25 (28.7)	22 (25.3)	0.015
Muslim (*n* = 207)	56 (27.1)	73 (35.3)	78 (37.7)	
Other (*n* = 12)	6 (50)	4 (33.3)	2 (16.7)	
Marital status				
Single (*n* = 38)	19 (50)	14 (36.8)	5 (13.2)	0.059
Married/In a relationship (*n* = 265)	82 (30.9)	87 (32.8)	96 (36.2)	
Separated/Divorced (*n* =3)	1 (33.3)	1 (33.3)	1 (33.3)	
Level of education				
No education (*n* = 23)	7 (30.4)	10 (43.5)	6 (26.1)	0.783
Primary and secondary education (*n* = 112)	33 (29.5)	38 (33.9)	41 (36.6)	
A levels/Higher vocational training (*n* = 93)	32 (34.4)	29 (31.2)	32 (34.4)	
University education (*n* = 78)	30 (38.5)	25 (32.1)	23 (29.5)	
Employment status				
Household chores (*n* = 95)	23 (24.2)	32 (33.7)	40 (42.1)	0.177
Employed (*n* = 144)	55 (38.2)	45 (31.3)	44 (30.6)	
Unemployed (*n* = 62)	24 (38.7)	23 (37.1)	15 (24.2)	
Student (*n* = 3)	0	1 (33.3)	2 (66.6)	
On sick leave (*n* = 2)	0	1 (50)	1 (50)	
Level of income per month				
<€500 (*n* = 12)	1 (8.3)	9 (75)	2 (16.7)	0.058
€5001–1000 (*n* = 101)	32 (31.7)	35 (34.7)	34 (33.7)	
€1001–2000 (*n* = 113)	34 (30.1)	36 (31.9)	43 (38.1)	
€2001–5000 (*n* = 74)	33 (44.6)	20 (27)	21 (28.4)	
>€5001 (*n* = 6)	2 (33.3)	2 (33.3)	2 (33.3)	
Number of children				
None (*n* = 117)	50 (42.7)	38 (32.5)	29 (24.8)	0.007
1–2 (*n* = 152)	47 (30.9)	51 (33.6)	54 (35.5)	
>2 (*n* = 37)	5 (13.5)	13 (35.1)	19 (51.4)	
Pre-pregnancy BMI				
Underweight	6 (46.2)	4(30.8)	3 (23.1)	0.510
Normal weight	48 (35.8)	41 (30.6)	45 (33.6)	
Overweight	31 (33)	36 (38.3)	27 (28.7)	
Obese	17 (26.2)	21 (32.3)	27 (41.4)	
Level of physical activity				
Low (*n* = 253)	76 (30)	94 (37.2)	83 (32.8)	0.004
Moderate (*n* = 53)	26 (49.1)	8 (15.1)	19 (35.8)	
High (*n* = 0)				
Trimester				
First trimester (*n* = 100)	39 (39)	35 (35)	26 (26)	0.023
Second trimester (*n* = 105)	39 (37.1)	36 (34.3)	30 (28.6)	
Third trimester (*n* = 101)	24 (23.8)	31 (30.7)	46 (45.5)	
Attendance at antenatal classes				
Yes (*n* = 119)	39 (32.8)	40 (33.6)	40 (33.6)	0.855
No (*n* = 176)	58 (33)	58 (33)	60 (34.1)	
Sometimes (*n* = 11)	5 (45.5)	4 (36.4)	2 (18.2)	

* Dietary pattern: the difference between the total recommended daily intake for each food group and the total daily intake score for all food groups was classified into three categories using two cutoff points (percentiles 33.3 and 66.6): Western pattern (−1.76, 0.13), *n* = 100 (32.8%); mixed pattern (1.04, 0.06), *n* = 104 (34%); and prudent pattern (3.82, 0.12), *n* = 102 (33.2%). y.o.: years old; BMI: Body mass index.

**Table 6:** Sociodemographic, lifestyle-related, and pregnancy-related variables and dietary patterns during pregnancy: ordinal linear regression model (*N* = 306) *.

Variables		Coef.	(95% CI)	*p*
Living in Melilla	Yes (*n* = 255)	Ref.	Ref.	Ref.
	No (*n* = 51)	0.368	(−0.269,1.005)	0.257
Age	<19 y.o. (*n* = 5)	Ref.	Ref.	Ref.
	20–39 y.o. (*n* = 289)	0.062	(−1.64,1.765)	0.943
	>40 y.o. (*n* = 12)	0.400	(−1.761,2.561)	0.717
Religion	Christian (*n* = 87)	Ref.	Ref.	Ref.
	Muslim (*n* = 207)	0.680	(0.038,1.322)	0.038
	Other (*n* = 12)	−0.389	(−1.594,0.816)	0.527
Marital status	Single (*n* = 38)	Ref.	Ref.	Ref.
	Married/In a relationship (*n* = 265)	0.297	(−0.438,1.031)	0.429
	Separated/Divorced (*n* = 3)	0.110	(−2.121,2.342)	0.923
Level of education	No education (*n* = 23)	Ref.	Ref.	Ref.
	Primary and secondary education (*n* = 112)	0.641	(−0.249,1.531)	0.158
	A levels/Higher vocational training (*n* = 93)	0.727	(−0.218,1.672)	0.131
	University education (*n* = 78)	0.877	(−0.161,1.916)	0.098
Employment status	Household chores (*n* = 95)	Ref.	Ref.	Ref.
	Employed (*n* = 144)	−0.442	(−1.116,0.231)	0.198
	Unemployed (*n* = 62)	−0.716	(−1.4,−0.032)	0.040
	Student (*n* = 3)	0.842	(−1.573,3.256)	0.494
	On sick leave (*n* = 2)	0.501	(−2.282,3.285)	0.724
Level of income per month	<€500 (*n* = 12)	Ref.	Ref.	Ref.
	€5001–1000 (*n* = 101)	−0.093	(−1.147,0.962)	0.863
	€1001–2000 (*n* = 113)	0.433	(−0.658,1.524)	0.437
	€2001–5000 (*n* = 74)	0.105	(−1.092,1.302)	0.864
	>€5001 (*n* = 6)	0.211	(−1.881,2.304)	0.843
Number of children	None (*n* = 117)	Ref.	Ref.	Ref.
	1–2 (*n* = 152)	0.497	(−0.022,1.017)	0.061
	>2 (*n* = 37)	1.002	(0.145,1.859)	0.022
BMI	Underweight	Ref.	Ref.	Ref.
	Normal weight	−0.097	(−1.26,1.067)	0.870
	Overweight	−0.425	(−1.633,0.783)	0.490
	Obese	0.281	(−0.936,1.499)	0.650
Level of physical activity	Low (*n* = 253)	Ref.	Ref.	Ref.
	Moderate (*n* = 53)	−0.197	(−0.875,0.481)	0.569
	High (*n* = 0)	-	-	-
Trimester	First trimester (*n* = 100)	Ref.	Ref.	Ref.
	Second trimester (*n* = 105)	−0.052	(−0.598,0.493)	0.851
	Third trimester (*n* = 101)	0.819	(0.25,1.387)	0.005
Attendance at antenatal classes	Yes (*n* = 119)	Ref.	Ref.	Ref.
	No (*n* = 176)	−0.342	(−0.861,0.178)	0.198
	Sometimes (*n* = 11)	−0.975	(−2.226,0.275)	0.126
/cut1		0.7719087		
/cut2		2.369885		

* Dietary patterns during pregnancy: prudent pattern score, Western pattern score, and difference in their scores: prudent pattern score minus Western pattern score; Ref.: reference group; Coef.: estimated coefficient; (95% CI): 95% confidence intervals of the estimated coefficient; *p*: *p*-value; /cut1: cut point 1; /cut2: cut point 2. y.o.: years old; BMI: Body mass index.
